# Colloidal Synthesis of CsX Nanocrystals (X = Cl, Br, I)

**DOI:** 10.3390/nano8070506

**Published:** 2018-07-07

**Authors:** Peter J. Shaw, Michaela Meyns, Yong Zuo, Albert Grau-Carbonell, Pavlos G. Lagoudakis, Martin D. B. Charlton, Sara Martí-Sánchez, Jordi Arbiol, Andreu Cabot, Antonios G. Kanaras

**Affiliations:** 1 School of Electronics and Computer Science, University of Southampton, Southampton SO17 1BJ, UK; ps1g12@soton.ac.uk (P.J.S.); mdbc@ecs.soton.ac.uk (M.D.B.C.); 2 Catalonia Institute for Energy Research (IREC), Jardins de les Dones de Negre 1, Sant Adrià de Besòs, 08930 Barcelona, Spain; michaela.meyns@awi.de (M.M.); yongzuo@irec.cat (Y.Z.); 3 Catalan Institute of Nanoscience and Nanotechnology (ICN2), CSIC and BIST, Campus UAB, 08193 Barcelona, Spain; a.graucarbonell@students.uu.nl (A.G.-C.); sara.marti@icn2.cat (S.M.-S.); arbiol@icrea.cat (J.A.); 4 Physics and Astronomy, Faculty of Physical Sciences and Engineering, University of Southampton, Southampton SO17 1BJ, UK; pavlos.lagoudakis@soton.ac.uk; 5 Skolkovo Institute of Science and Technology Novaya St., 100, Skolkovo 143025, Russian Federation; 6 ICREA, Pg. Lluís Companys 23, 08010 Barcelona, Spain

**Keywords:** cesium halide, colloidal synthesis, monodispersed nanocrystals, processability

## Abstract

A facile colloidal synthesis of highly ionic cesium halide nanocrystals is reported. Colloidal nanocrystals of CsI, CsCl and CsBr with unprecedentedly small dimensions are obtained using oleylammonium halides and cesium oleate as precursors. The ease and adaptability of our method enables its universalization for the formation of other highly ionic nanocrystals.

## 1. Introduction

The colloidal synthesis of nanocrystals has reached a degree of control over composition, size and shape that allows precise manipulation of physical and chemical properties of a wide range of materials. Despite this fact, highly ionic compounds, such as alkali halides, are a class of materials that easily form macroscopic crystals of high purity and quality, but pose a significant challenge in regard to nanocrystal formation. The strong ionicity of the atomic bonds renders alkali halide crystals highly soluble in polar solution and sensitive to humidity, which poses a major obstacle for nanocrystal synthesis.

The most ionic examples of alkali halides and, in fact, the most ionic binary compounds available are the halides of cesium, CsX (X = F, Cl, Br, I). These materials exhibit wide band-gaps, with energies ranging from 6.1 to 8.3 eV at ambient conditions [1,2,3], while changing to metallic phase at high pressures [4]. A peculiarity of CsI and CsCl is their ability to exhibit an optical color due to the phenomenon of halide vacancies, an effect that is still being actively investigated in other alkali halide salts [1,3,5].

Owing to their simplicity, cesium halides are excellent model systems to validate methodologies and theories directed to produce and describe ionic crystals [6]. Additionally, cesium halides find multiple applications, ranging from their use as IR-transparent CsBr and CsI beam splitters to the use of CsI as an efficient photocathode at extreme ultraviolet and X-ray frequencies [7,8]. Recently, CsBr has been used to passivate the surface of cesium lead bromide perovskite nanocrystals [9]. Similarly, CsCl layers with nanometer thickness have seen applications as an efficient electron injection layer in light emitting diodes [10,11]. CsX nanostructures and nanocrystals have also been employed to improve emission currents from carbon nanotubes, detectionizing radiation for medical applications, and enhance the process of photolithographic etching [12,13,14].

The difficulties surrounding the synthesis of CsX nanocrystals are rooted in the intrinsic ionicity of the binary salt system. For instance, with an electronegativity difference of 2.4, crystals of CsCl are very sensitive to moisture in the atmosphere, rapidly changing from single cubes to polycrystalline dendrites in ambient conditions [15].

Among the successful methods that have been applied in the synthesis of alkali halides are high temperature vapor–liquid–solid techniques, enabling the production of either nanowires [16] or nanoparticles [12], a water-based reverse micellar synthetic method [17] and electrospray techniques [18]. However, none of these methods have shown the versatility usually provided by colloidal nanocrystal synthesis in organic solutions. An important step in this direction was made by applying a colloidal hot injection protocol with the halide source provided by a metallic salt, yielding CsX microcrystals with dimensions in the range of 100–500 nm [19]. Utilization of a metallic salt is a straightforward solution; however, the introduction of an additional metallic counter-ion into the protocol may produce undesirable by-products or introduce impurities to the nanocrystal.

An increased understanding of the in situ formation of long-chained but ionic organic ligands and their role in the dynamic stabilization of nanocrystals with relatively high polarity motivated us to investigate the possibility of employing them as stabilizers for CsX and at the same time provide the desired halide in form of the counter-ion [20].

In the following, we detail a facile synthetic protocol to produce colloidal CsX nanocrystals with sizes on the order of tens of nanometers based on this metal-free approach. Furthermore, we explore the effect of variations in the halide source on the size and shape of the obtained nanocrystals.

## 2. Materials and Methods

### 2.1. Chemicals

Cesium acetate (CsOAc, 99.9%), 1-octadecene (ODE, 90%), hydrobromic acid (48% wt.), hydrochloric acid (37% wt.), hydroiodic acid (57% wt.) and hexane were purchased from Sigma Aldrich (Madrid, Spain). Oleic acid (OLA, 90%) and iodine (>99.5%) were bought from Fluka (Bucharest, Romania), while oleylamine (80–90% C18) was produced by Acros Organics (New Jersey, USA). Absolute ethanol, ethyl acetate (p.A.) and toluene (p.A.) were purchased from Fisher Scientific (Madrid, Spain).

### 2.2. Synthesis of Oleylammonium Halide

In a three-neck flask, 9.9 g (37 mmol, based upon a purity of 80%) of oleylamine was mixed with 100 mL of ethanol. This mixture was cooled to 0 °C in an ice–water bath; then, 76 mmol of either HCl (6.3 mL, 37% wt.) or HBr (8.6 mL, 48% wt.) or HI (10 mL, 57% wt.) was added dropwise. After this addition, the solution was left to be stirred under an argon atmosphere for 20 h [21]. The solvent was then removed under reduced pressure in a rotary evaporator. The obtained crude product was dispersed in a minimum of ethyl acetate and sonicated for up to 1 min. The resulting solution was then centrifuged at 7000 rpm for 5 min. This process was repeated until a colorless supernatant and a white powder-like precipitate were obtained.

In the case of oleylammonium iodide, oily by-products prevented the precipitation of the oleylammonium compound at room temperature, so an ethanol–liquid nitrogen bath was used to achieve precipitation. Once the precipitate was separated from the oily liquid, it could then be washed in the same way as the other halide compounds.

### 2.3. Synthesis of Cesium Oleate Precursor

CsOAc (0.884 g, 4.61 mmol), ODE (40 mL), and OLA (2.5 mL) were placed into a three-neck flask and degassed under vacuum (<10^−2^ mbar) for 1 h at 120 °C. After the degassing stage, the flask was kept under a flow of argon. The temperature of the solution was raised to 150 °C until the solution became clear, indicating the formation of the soluble cesium oleate [22,23]. For storage under an inert atmosphere, the temperature of the solution was lowered to room temperature, or the solution was held at 120 °C for the hot injection as detailed below.

### 2.4. Synthesis of CsX Nanocrystals Using Oleylammonium Halide

ODE (5.0 mL), OLA (0.5 mL, 1.4 mmol for 90% purity), and oleylammonium halide (46 μmol–14 mg oleylammonium chloride; 16 mg oleylammonium bromide; 18 mg oleylammonium iodide) were placed in a three-neck flask and degassed under vacuum (<10^−2^ mbar) at 120 °C for 1 h. The flask was then placed under an argon flow. Once a clear solution was obtained, the temperature of the solution was raised to 120 °C, and 43 μmol (0.4 mL) of the as-prepared cesium oleate precursor held at 120 °C was swiftly injected. After 5 s, the flask containing the crude CsX solution was rapidly cooled to room temperature using an ice bath. Here, care was taken to avoid freezing the solution, as ODE has a melting point of 14 °C.

### 2.5. Synthesis of CsX Nanocrystals Using Novel Precursors

Identical to the synthetic protocol using oleylammonium halide, with the following reactant substitutions in place of oleylammonium halide:

- In the case of cetyltrimethylammonium bromide (CTAB), CTAB (18 mg, 49 μmol) was used.
- In the case of iodine, iodine (24 mg, 0.19 mmol) was dissolved in 0.5 mL (80–90%) of oleylamine by stirring and this mixture was used.
- In the case of CuBr_2_, CuBr_2_ (40 mg, 0.188 mmol) was dissolved in 0.5 mL (80–90%) of oleylamine by stirring at room temperature and a lower reaction temperature of 90 °C was used.

### 2.6. Purification of Crude CsX Nanocrystals

The crude solution of CsX was subjected to centrifugation at 8000 rpm for 15 min. After this, the supernatant was discarded, and the precipitate dispersed in 5 mL of the organic solvent, and was then stored in an argon-flushed and sealed vial.

### 2.7. Characterization

Low resolution transmission electron microscopy (TEM) (Carl Zeiss, Jena, Germany) was conducted on a ZEISS LIBRA 120, operating at 120 kV. Carbon-coated TEM grids from Ted-Pella were used as substrates. High resolution TEM (HRTEM) and scanning TEM (STEM) images were recorded using an FEI Tecnai F20 TEM microscope (FEI company, USA), equipped with a high angle annular dark field (HAADF) detector (FEI company, USA) and operated at 200 kV. Energy-dispersive X-ray spectroscopy (EDS) spectra were obtained in the HAADF-STEM mode. The EDS spectrum of nanocrystals obtained with CuBr^2^ was gathered using an Auriga Zeiss field-emission scanning electron microscope (SEM) (Carl Zeiss, Jena, Germany) at 5.0–20 kV on a Si wafer. Powder X-ray diffraction (XRD) patterns were obtained with Ni-filtered (2 μm thickness) Cu Kα1 (λ = 1.5406 A) radiation in a reflection geometry on a Bruker-AXS D8 Discover diffractometer (Bruker, Karlsruhe, Germany) operating at 40 kV and 40 mA.

## 3. Results and Discussion

To obtain cesium halide nanocrystals, we injected a hot solution of cesium oleate into a solution of the desired halide precursor and oleic acid in octadecene [22,23]. For optimum solubility and to avoid the use of further metal cations, we employed oleylammonium halides, prepared from oleylamine and the respective mineral acid [21], as a combined source of anions and a stabilizer. The temperature of both solutions was kept at 120 °C and the reaction was left to proceed for 5 s before cooling down in an ice–water bath. During cooling, a white precipitate was formed. After centrifugation, this precipitate could be re-dispersed in non-polar organic solvents, such as hexane or toluene, obtaining a turbid white solution, with the relative turbidity increasing concomitantly with anion size. The overall yield of the reaction and collection of the precipitate was ca. 90%.

**Figure 1:**
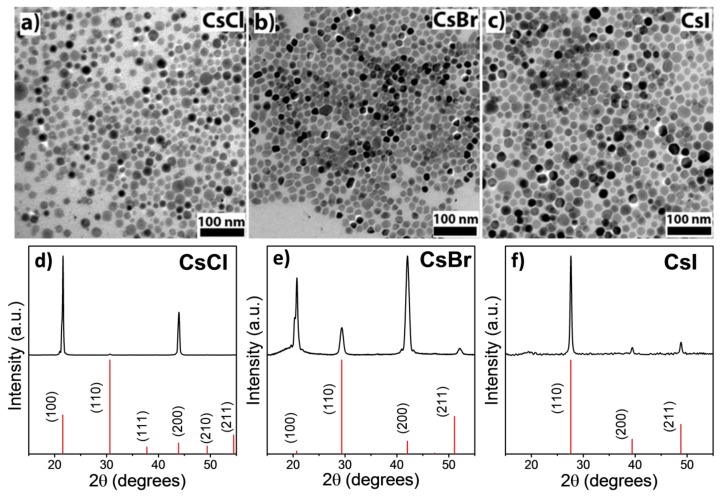
(**a**)–(**c**) TEM micrographs of obtained CsX nanocrystals, and (**d**)–(**f**) corresponding XRD patterns. Reference patterns for CsX are also included as references: CsCl (00-005-0607); CsBr (00-005-0588); CsI (00-006-0311).

Figure 1a–c shows TEM micrographs of the quasi-spherical nanocrystals obtained by the above-described procedure. Size distribution histograms can be found in Figure S1 in the ESI. Under identical conditions, the diameter of the nanocrystals slightly increased from CsCl (17 ± 3 nm) to CsI (20 ± 5 nm), which was concomitant with the lattice parameters and the anion size. In agreement with their wide optical transparency in bulk dimensions, we did not observe optical activity in these nanocrystals.

The stability of the produced colloidal CsI and CsBr samples was noted to be superior to that of the CsCl nanocrystals. CsI and CsBr nanocrystals retained their physical structures after weeks in the colloidal suspension and storage under ambient conditions. Similarly, they demonstrated a stable structure during low resolution TEM analysis. On the other hand, within a matter of days, CsCl samples underwent ripening and aggregation as observed by TEM analysis. This relative instability has been previously observed for CsCl microcrystals, and it was attributed to the higher lattice energy of CsCl that may favor rearrangement [15,24]. We have reached a similar hypothesis, that is, the difference in stability was the result of a higher electronegativity value for chloride ions compared to that of bromide and iodide, as well as the documented hygroscopic nature of CsCl destabilizing the ionic crystal and inducing a morphological change in the presence of humidity [15,25]. Morphology changes are especially notorious when stabilizing ligands begin to degrade or evaporate under the influence of the electron beam.

XRD patterns (Figure 1d–e) showed the crystallographic structure of all the nanocrystal samples matched with the cubic crystal structure, *Pm3m* space group, of bulk CsX [26,27]. While the relative intensities of the recorded pattern matched well with the reference for CsI, both CsCl and CsBr showed a higher contribution of the (100) and (200) diffraction planes compared to the standard. This effect may be ascribed to a preferential orientation of the nanocrystals on the substrate. As evident from the comparison with the diffractogram of pure Cs-oleate (Figure S5), the wide, flat signal below 20 degrees resulted from small residues of the precursor that were difficult to be removed without destroying the structure.

**Figure 2:**
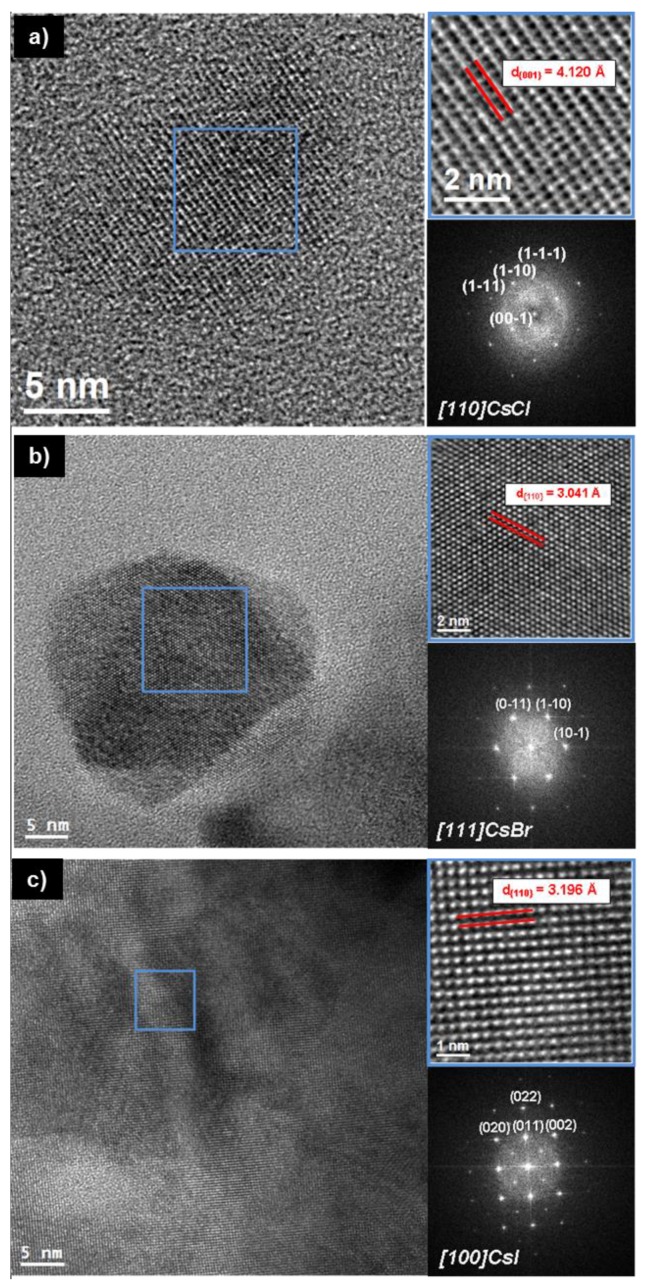
HRTEM and power spectrum analysis of CsX nanocrystals. (**a**) CsCl nanocrystal in a view along the [110] zone axis. (**b**) CsBr nanocrystal in a view along the [111] zone axis. (**c**) CsI nanocrystal in a view along the [100] zone axis. Despite the morphological changes induced by the inelastic interaction between electron beam and sample, due to the high ordering in strongly ionic compounds, it is possible to obtain lattice fringes.

High resolution TEM (HRTEM) power spectral analysis confirmed all of the nanocrystals to be of the cubic Pm3m space group (Figure 2). EDS analysis also showed stoichiometric ratios of CsX of approximately 1:1 in all cases with slight excess of Cs in the case of CsBr (Figures S6–S8). Long electron beam exposure during TEM analysis led to considerable degradation of the nanocrystals, a common problem in alkali halides due to defect generation through ionization caused by the incident electron beam [28]. The degradation was observed to initially result in the hollowing of the individual nanocrystals (Figure S9). While the exact mechanism remains unclear, it can be presumed to be related to halogen ejection observed in other alkali halide nanocrystals under powerful irradiation [29].

Besides oleylamonium halides, we studied other sources for halide introduction, including the use of cetyltrimethylammonium bromide (CTAB) and metallic iodine (I_2_) as a halide source for CsBr and CsI synthesis, respectively. Additionally, a metal salt, CuBr_2_, was also tested.

**Figure 3:**
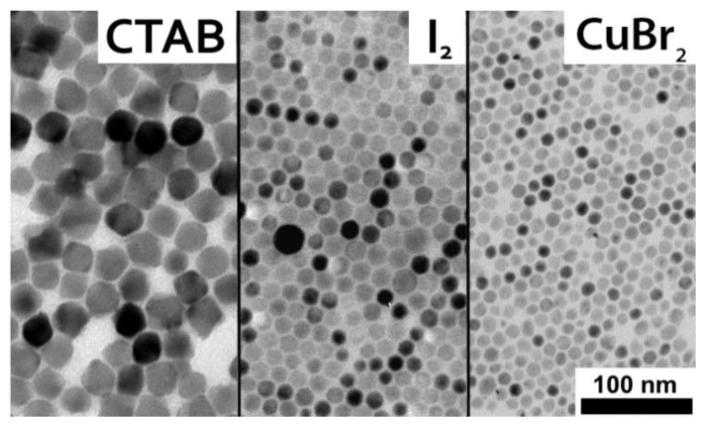
TEM micrographs of CsBr and CsI nanocrystals synthesized using cetyltrimethylammonium bromide (CTAB), I_2_, and CuBr_2_, respectively.

CTAB produced larger (29 ± 4 nm) and more faceted CsBr nanocrystals than those obtained with oleylammonium bromide (Figure 3 and Figure S10). The reason that CTAB led to larger crystals may be a change in the interaction between the ions, leading to altered nucleation kinetics or a weaker surface protection of the growing nanocrystals due to the higher steric demand of the trimethylammonium head group, compared to the simple primary ammonium group in oleylammonium ions.

The use of metallic I_2_ dissolved in oleylamine resulted in similar CsI nanocrystals to those produced by oleylammonium iodide, but with smaller sizes of 17 ± 4 nm (Figure 3 and Figure S11). This difference was reasoned to be due to the abundance of oleylamine added during synthesis, resulting in a faster surface passivation of nucleated nanocrystals after halide delivery.

CuBr_2_ produced small CsBr nanocrystals with a narrow size distribution. However, it was found to leave a residual copper content (Figure 3 and Figure S12, Table S2). This shows the issues arising when a metallic salt for nanoparticle synthesis is utilized, as counter-ions are released into the solution upon halide delivery and contaminate the synthesized nanocrystals.

## 4. Conclusions

To summarize, we have presented a method for the synthesis of colloidal CsX nanocrystals with dimensions on the order of tens of nanometres, avoiding inorganic salts in the reaction. The nanocrystals were shown to have narrow size distributions and a well-defined cubic Pm3m structure. We also demonstrated the flexibility of this method by testing other halide sources, showing that the CsX nanocrystals possess a degree of processability that makes them ripe for future investigations, for example, in terms of their effect on electron extraction from adjacent metals.

## Supplementary Materials

The following are available online http://www.mdpi.com/2079-4991/8/7/506/s1. Figure S1: Size Distribution of obtained CsX nanocrystals; Figure S2: Full diffractogram of CsCl; Figure S3: Full diffractogram of CsBr; Figure S4: Full diffractogram of CsI; Figure S5: Diffractogram of cesium oleate; Figure S6: EDX spectrum acquired on CsCl nanocrystals; Figure S7: EDX spectrum of the shown area (inset) consisting of CsBr nanocrystals.; Figure S8: EDX spectrum of the shown area on the CsI nanocrystals (inset); Figure S9: Evolution of a CsBr nanocrystal under HRTEM beam; Figure S10: Size Distribution of obtained CsBr nanocrystals from a precursor of CTAB; Figure S11: Size Distribution of obtained CsI nanocrystals from a precursor of I_2_; Figure S12: EDS spectrum of obtained CsBr nanocrystals from a precursor of CuBr_2_; Table S1: SEM-EDX data of CsI which is more stable in this type of quantification experiment; Table S2: Elemental Cs, Cu and Br content of spectra A and B of the sample obtained from CuBr_2_ determined by EDS
