# Supplementary material for: Colloidal Synthesis of CsX Nanocrystals (X = Cl, Br, I)
Source: Nanomaterials (Basel). 2018 Jul 7;8(7):506. doi: 10.3390/nano8070506 (PMC6070971; doi:10.3390/nano8070506)
Supplement: Supplementary file 1 [file nanomaterials-08-00506-s001.pdf]

Supporting Information

Colloidal Synthesis of CsX Nanocrystals (X = Cl, Br, I)

Peter J. Shaw [1,†], Michaela Meyns [2,†], Yong Zuo [2], Albert Grau-Carbonell [3], Pavlos G. Lagoudakis [4, 6], Martin D. B. Charlton [1], Sara Martí-Sánchez [3], Jordi Arbiol [3,5], Andreu Cabot [2,5,*] and Antonios G. Kanaras [4, *]

1 School of Electronics and Computer Science, University of Southampton, Southampton SO17 1BJ, UK; ps1g12@soton.ac.uk (P.J.S.); mdbc@ecs.soton.ac.uk (M.D.B.C.)

2 Catalonia Institute for Energy Research (IREC), Jardins de les Dones de Negre 1, Sant Adrià de Besòs, Barcelona 08930, Catalonia, Spain; michaela.meyns@awi.de(M.M.); yongzuo@irec.cat (Y.Z.)

3 Catalan Institute of Nanoscience and Nanotechnology (ICN2), CSIC and BIST, Campus UAB, Bellaterra, 08193 Barcelona, Catalonia, Spain; a.graucarbonell@students.uu.nl (A.G.C.); sara.marti@icn2.cat (S.M.S.); arbiol@icrea.cat (J.A.)

4 Physics and Astronomy, Faculty of Physical Sciences and Engineering, University of Southampton, Southampton SO17 1BJ, UK; pavlos.lagoudakis@soton.ac.uk (P.G.L.)

5 ICREA, Pg. Lluís Companys 23, 08010 Barcelona, Catalonia, Spain

6 Skolkovo Institute of Science and Technology Novaya St., 100, Skolkovo 143025, Russian Federation

* Correspondence: acabot@irec.cat (A.C.); a.kanaras@soton.ac.uk (A.G.K.); Tel.: +34 625615115 (A.C.) & +023 80592466 (A.G.K.)

† These authors contributed equally to this work.

1 Size Distribution Analysis

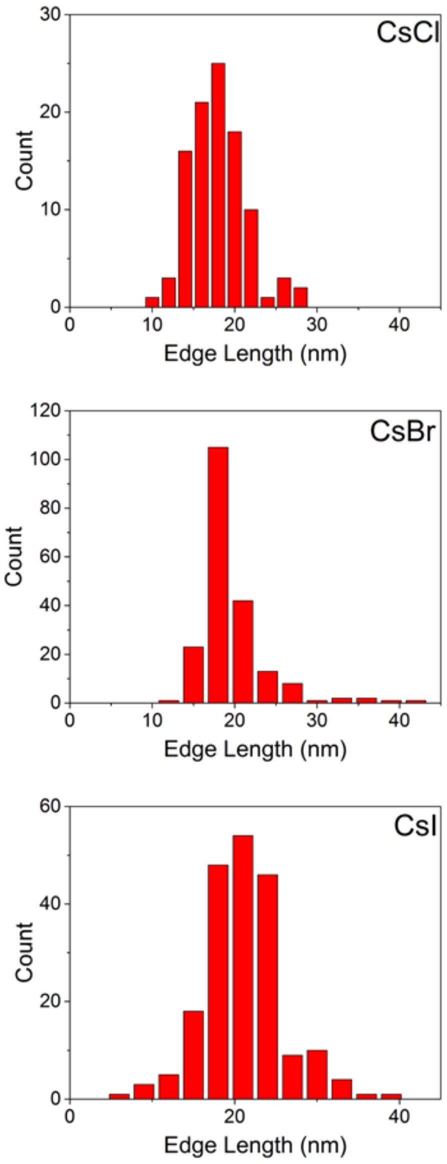

Figure S1. Size Distribution of obtained CsX nanocrystals.

2 XRD Analysis

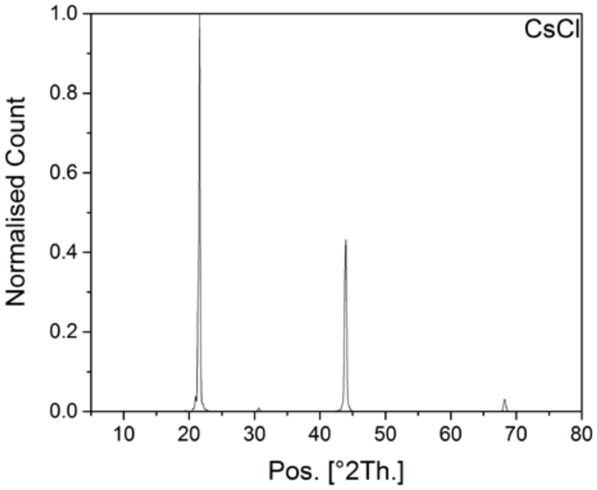

Figure S2. Full diffractogram of CsCl.

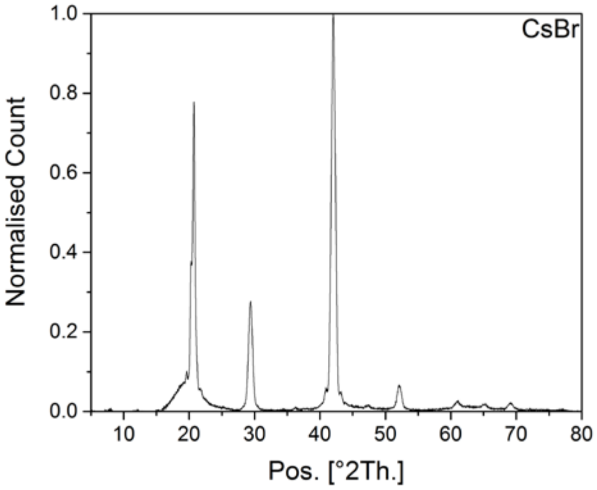

Figure S3. Full diffractogram of CsBr.

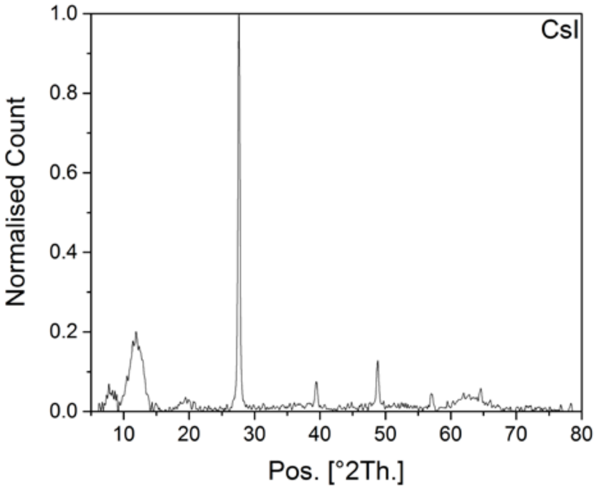

Figure S4. Full diffractogram of CsI.

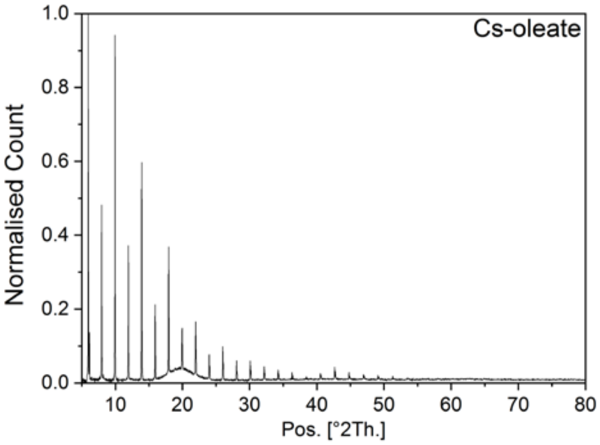

Figure S5. Diffractogram of cesium oleate.

3 EDX Analysis

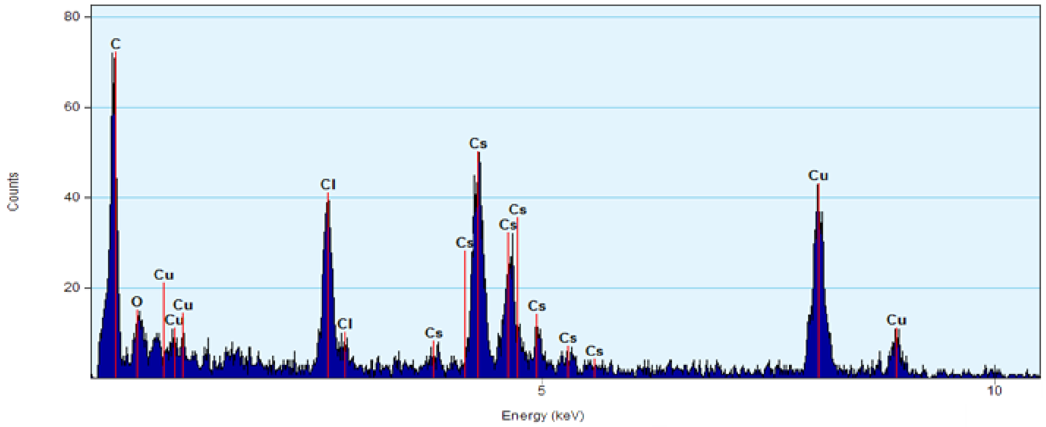

Figure S6. EDX spectrum acquired on CsCl nanocrystals. Both cesium and chlorine were identified. Peaks of copper and carbon arise from the supporting grid. The determined atomic percentages are (45±1) % Cl and (55±1) % Cs, which is stoichiometric in the range of accuracy of TEM-EDX. A slight excess would anyhow comply with an outer layer of Cs atoms.

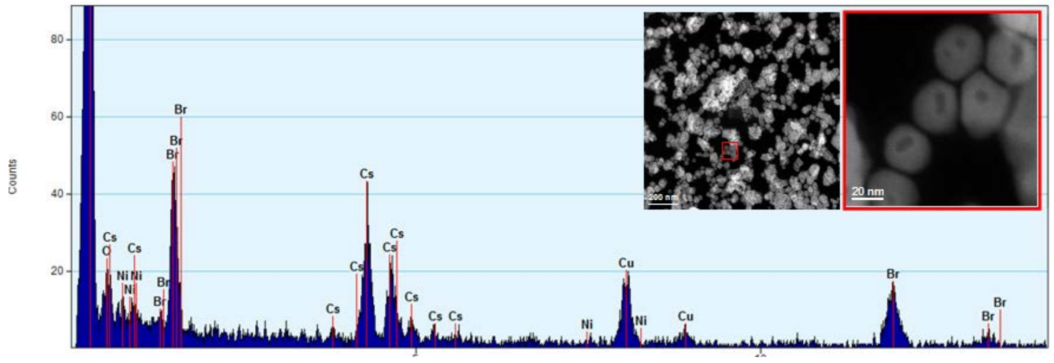

Figure S7. EDX spectrum of the shown area (inset) consisting of CsBr nanocrystals. The signals of cesium and bromide were detected. Peaks from nickel, copper and carbon were a result of the supporting grid. The oxygen peak could arise from the residual presence of organic compounds. The content of elements was (40±3) % Br and (60±3)% Cs in at.%, indicating an excess of Cs, which can be explained by Cs as terminating layer and, in line with XRD, a small residue of Cs-oleate.

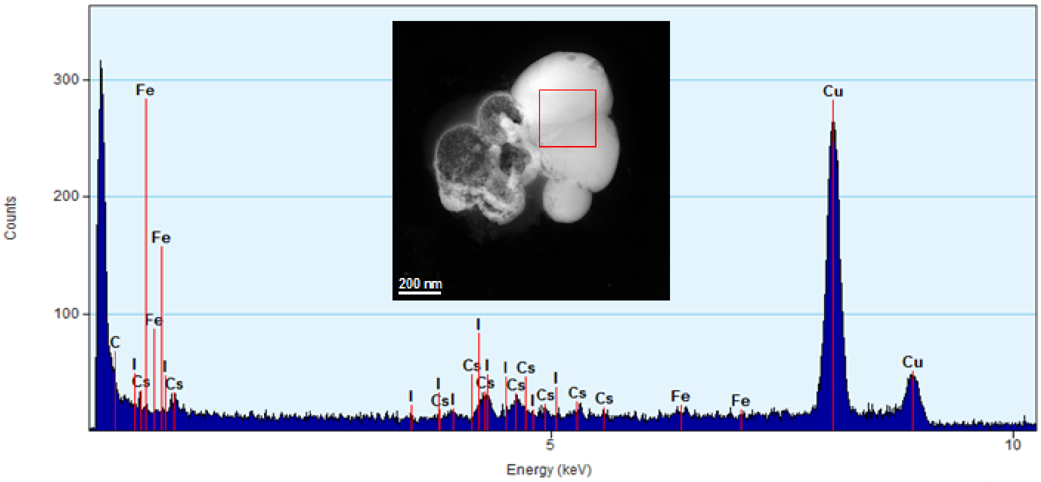

Figure S8. EDX spectrum of the shown area on the CsI nanocrystals (inset). The signals of cesium and iodine were detected. Peaks from iron, copper and carbon were a result of the supporting grid. We could not measure enough datapoints to quantify the atomic masses by TEM-EDX due to the rapid evaporation.

Table S1: SEM-EDX data of CsI which is more stable in this type of quantification experiment (not-rounded values for I and Cs are both close to 14.5%).

Element	Weight%	Weight% Sigma	Atomic%
C K	18	0.7	71
I L	41	0.6	15
Cs L	41	0.6	14

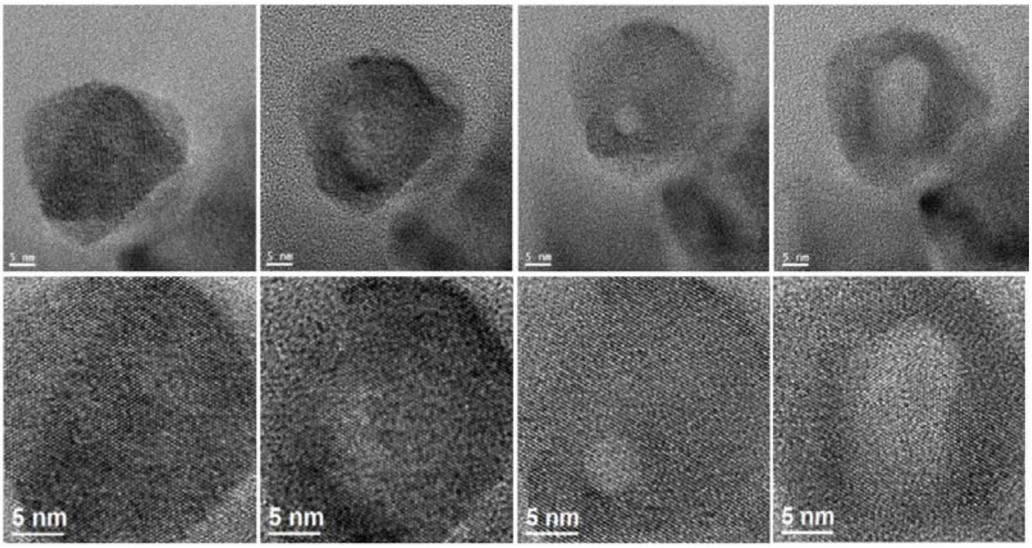

Figure S9. Evolution of a CsBr nanocrystal under HRTEM beam. A hollow space appeared inside the particle and grew over time (increasing time exposure from left to right). The total time exposure between the first and last acquisition was approximately 1.5 min.

4 Other Halide Sources

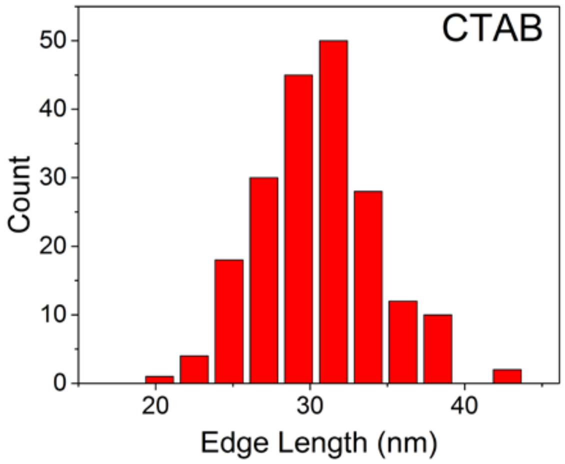

Figure S10. Size Distribution of obtained CsBr nanocrystals from a precursor of CTAB.

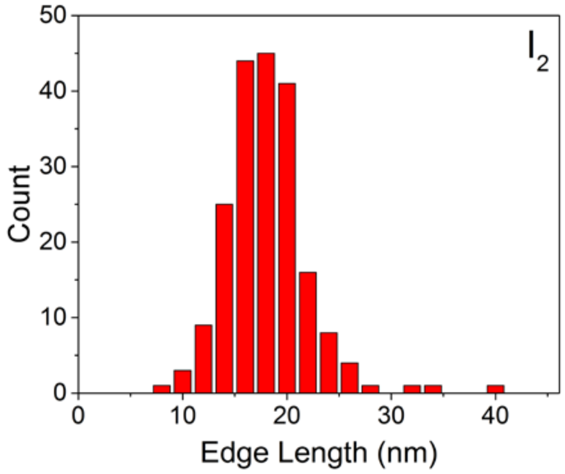

Figure S11. Size Distribution of obtained CsI nanocrystals from a precursor of I_2.

A

B

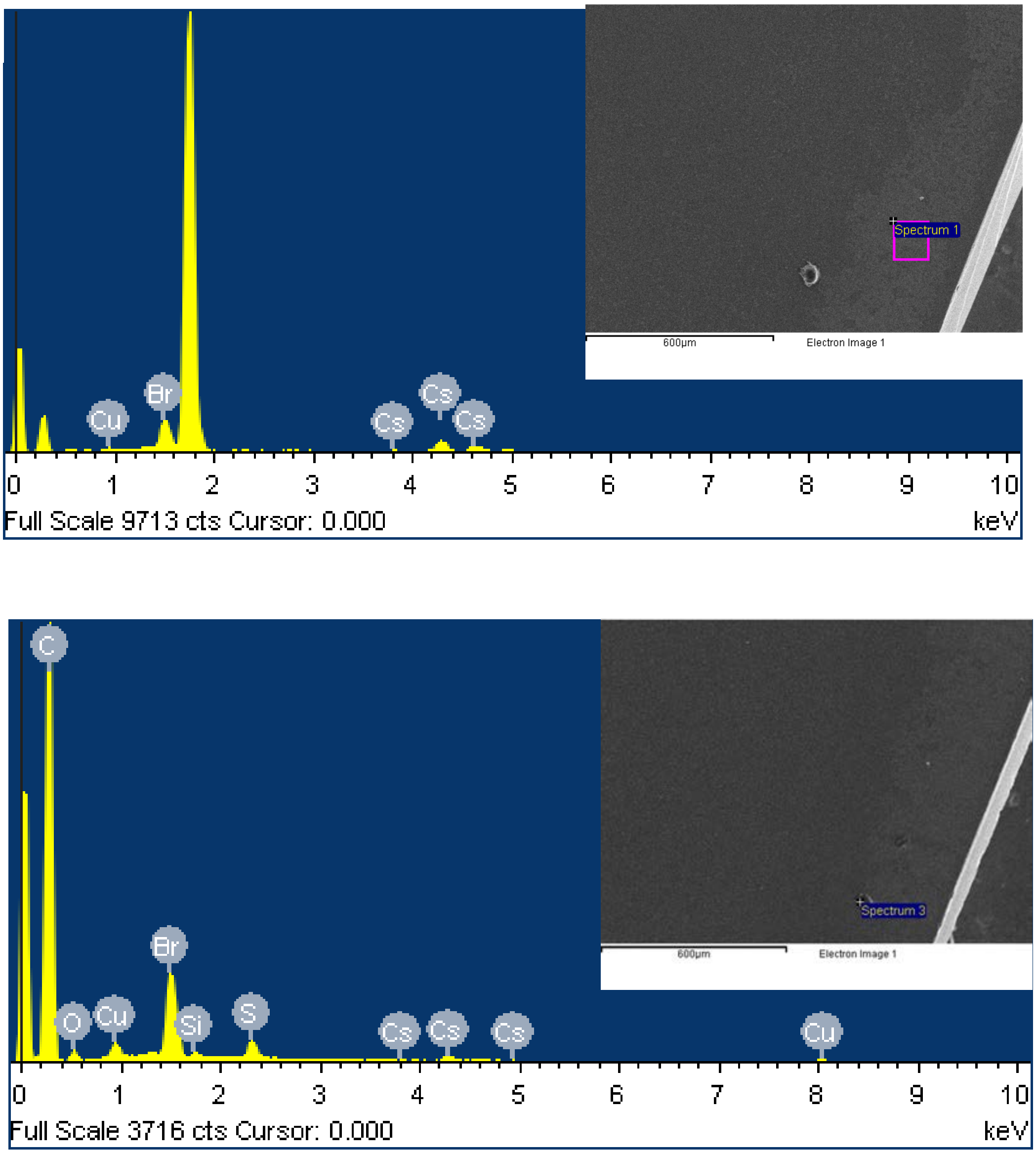

Figure S12. EDS spectra of obtained CsBr nanocrystals from a precursor of $CuBr_2$. Peaks of Si arise from the supporting Si wafer. Peaks of Cs, Br, and Cu can be seen, spots with varying Cu content are observed.

Table S2: Elemental Cs, Cu and Br content of spectra A and B of the sample obtained from $CuBr_2$ determined by EDS.

**Element**	**Weight%**	**Atomic%**
Spectrum A		
Cu K	2	4
Br L	61	71
Cs L	37	26
Spectrum B		
Cu K	9	11
Br L	81	83
Cs L	10	6
